# Canagliflozin-associated diabetic ketoacidosis: a case report

**DOI:** 10.1080/24734306.2017.1331604

**Published:** 2017-05-25

**Authors:** Peter R. Chai, Caitlin Bonney, Eike Blohm, Edward W. Boyer, Kavita M. Babu

**Affiliations:** a Department of Emergency Medicine, Brigham and Women's Hospital, Boston, MA, U.S.A; b Department of Emergency Medicine, University of Massachusetts Medical School, Worcester, MA, U.S.A; c Division of Medical Toxicology, Department of Emergency Medicine, University of Massachusetts Medical School, Worcester, MA, U.S.A

**Keywords:** Canagliflozin, SGLT inhibitors, diabetic ketoacidosis, adverse events, diabetes

## Abstract

Canagliflozin is a novel sodium-glucose cotransporter-2 (SGLT-2) inhibitor approved for the management of diabetes. We report the presentation and management of two cases of canagliflozin associated diabetic ketoacidosis (DKA) and discuss the mechanism of canagliflozin associated DKA. Patient 1, a 55 year old woman maintained on canagliflozin for diabetes mellitus II presented to the emergency department (ED) with 24 hours of nausea and vomiting. She was diagnosed with DKA featuring hypotension, hyperglycemia, ketosis and acidosis. A second 54 year old man also maintained on canagliflozin for diabetes mellitus I presented to the ED with 24 hours of nausea and vomiting. He was diagnosed with DKA with similar manifestations as patient 1. Both patients underwent massive volume resuscitation and intravenous insulin therapy with resolution of ketosis and acidosis. By inhibiting SGLT-2, canagliflozin promotes glucosuria, which in turn can produce up to a 10% decrease in total plasma volume rendering patients maintained on canagliflozin susceptible to dehydration. Inhibition of SGLT-2 also leads to glucagon secretion, which in the volume deplete individual, can exacerbate DKA. Physicians should be aware of the rapid onset of DKA in patients maintained on canagliflozin after just minor additional fluid losses.

## Introduction

Canagliflozin is an oral sodium–glucose cotransporter-2 (SGLT-2) inhibitor approved by the Food and Drug Administration (FDA) in 2013 to improve glycemic control in patients with type 2 diabetes (T2DM). Common adverse events associated with canagliflozin include genitourinary infections, hypoglycemia, polydipsia, and urinary frequency [1]. Since its introduction, 195 cases of diabetic ketoacidosis (DKA) associated with canagliflozin have been reported to the FDA. In 2015, the FDA issued a warning that SGLT-2 inhibitors including canagliflozin may increase the risk of DKA [2]. We report two patients diagnosed with canagliflozin-associated DKA, review SGLT pathophysiology and pharmacology, and propose a mechanistic rationale for canagliflozin-associated DKA.

## Case 1

A 55-year-old woman with history of T2DM on canagliflozin 300 mg daily developed nausea, vomiting, and polyuria over 24 hours. Vital signs showed tachycardia and hypotension (Table 1). On examination, she had dry mucous membranes and epigastric abdominal pain. Lab values revealed acidosis with an anion gap of 32 mmol/L, and acute kidney injury (Table 1). Treating clinicians diagnosed DKA based upon serum beta hydroxybutyrate (BHB) concentration of 12.43 mmol/L although her serum glucose concentration was 366 mg/dL (20.3 mmol/L). Her lipase concentration of 165 U/L suggested acute pancreatitis. She received continuous insulin infusion and intravenous normal saline resuscitation. In the intensive care unit, her anion gap normalized. She was transitioned to metformin and glipizide and discharged home on hospital day 3.

## Case 2

A 54-year-old man with type 1 diabetes mellitus (T1DM) on insulin (humalog 25/35 units subcutaneous every AM/PM), albiglutide 50 mg, and canagliflozin 300 mg PO daily developed abdominal pain, nausea, and vomiting. Vital signs showed tachycardia and tachypnea. His examination was significant for dry mucous membranes and epigastric tenderness. Laboratory analysis revealed acidosis with an elevated anion gap of 27 mmol/L (Table 1). A BHB concentration of 15.35 mmol/L with a glucose concentration of 327 mg/dL (18.2 mmol/L) led to a diagnosis of DKA. He underwent volume resuscitation and an intravenous insulin infusion until the anion gap had normalized. The patient improved and returned home after 3 days of hospitalization.

## Discussion

Our cases describe acute onset DKA in patients with T1DM and T2DM on canagliflozin. The onset of DKA in both patients followed acute gastrointestinal illness, suggesting volume contraction from canagliflozin in addition to insensible losses contributed to the development of ketoacidosis. Recognition of canagliflozin-induced volume contraction guided aggressive intravenous fluid resuscitation in both patients. Patients with diabetes on canagliflozin may present with rapid onset DKA associated with minor inciting events. Serum glucose concentrations are often only modestly elevated. Early intervention with intravenous fluids and insulin infusions can help correct metabolic derangements associated with canagliflozin-associated DKA.

Canagliflozin inhibits SGLT-2, thereby inducing glucosuria and decreasing serum glucose. Three types of sodium–glucose cotransporters are expressed in the proximal renal tubule, pancreatic alpha cells, small intestine mucosa, and hypothalamus [2,3] (Table 2). SGLT-1 and -2 are found in the proximal renal tubules where they reabsorb 90% of filtered glucose [3]. SGLT-2 is also found in pancreatic alpha cells where antagonism induces glucagon release [4].

SGLT-2 facilitates glucose reabsorption into renal tubule epithelial cells via active sodium cotransport maintained by sodium/potassium ATPases [3]. Rising plasma glucose levels increase the amount of glucose reabsorbed by SGLT to a serum glucose threshold of approximately 200 mg/dL, where SGLT is saturated, inducing glucosuria in healthy individuals [3,5]. Individuals with diabetes mellitus develop chronic hyperglycemia and dysregulation of the protective mechanism of SGLT. As renal tubular cells are exposed to elevated blood glucose levels, SGLT is upregulated by as much as 20%–40%, resulting in escalating glucose absorption [6,7].

Canagliflozin is a synthetic thiopene with high affinity for SGLT-2 [2]. Canagliflozin diffuses into the renal filtrate where it binds the glucose transport site of SGLT-2, inhibiting glucose reabsorption and promoting glucosuria [8]. Canagliflozin-induced glucosuria results in longterm calorie wasting, weight loss, and improved glycemic control [9]. Inhibition of SGLT-2 also induces glucagon secretion in alpha cells of the pancreas, minimizing the potential for hypoglycemic events when prescribed as sole therapy for DM [4].

Adverse effects associated with canagliflozin in initial safety trials include increased incidence of genitourinary infections, increased serum creatinine, hypoglycemia when administered with sulfonylureas or insulin, and hyperkalemia in patients with chronic kidney disease [10]. Serious adverse events included DKA and pancreatitis [10]. One hundred and ninety five cases of canagliflozin-associated DKA have been reported to the FDA since 1997 [11]. Data regarding the increased risk for the development of DKA in canagliflozin therapy are inconclusive [1,12]. Predisposing factors for canagliflozin-associated DKA include T1DM, concurrent insulin therapy, and an inciting illness such as gastroenteritis. Because canagliflozin promotes glucosuria, episodes of DKA can present with lower blood glucose levels than expected [13]. Canagliflozin is not FDA-approved for use in T1DM, and studies investigating its role as an addition to insulin therapy in patients with T1DM suggest that it may increase the risk of DKA [12].

We suspect that canagliflozin-associated DKA is due to four mechanisms. First, in the setting of gastrointestinal illness or disease states with high insensible loss, the additional volume contraction from glucosuria due to SGLT-2 inhibition can lead to ketoacidosis. This effect is likely exaggerated during initiation of canagliflozin therapy with pharmacodynamic studies showing up to a 10% total plasma volume contraction in the initial week of therapy [11]. Second, antagonism of SGLT-2, while promoting glucosuria, also stimulates glucagon secretion, contributing to hyperglycemia which can be significant in the volume-deplete patient [4]. Third, glucosuria increases both plasma glucose and insulin sensitivity. The elevated glucagon–insulin ratio in the setting of decreased carbohydrate availability stimulates lipolysis, producing ketoacidosis. Fourth, acute prerenal azotemia downregulates SGLT-2 expression in the proximal tubules, creating an environment for nonspecific binding of canagliflozin to other sodium–glucose cotransporters that contribute to hyperglycemia [14].

## Why should an emergency physician be aware of this?

Physicians prescribing SGLT-2 inhibitors should recognize the association of canagliflozin with DKA. Canagliflozin-induced glucosuria may produce falsely reassuring blood glucose concentrations in patients with increased insensible losses. Volume contraction from glucosuria may contribute to DKA, the risk of which is more pronounced in patients who have recently initiated canagliflozin therapy. Physicians evaluating patients on canagliflozin with gastrointestinal distress should be vigilant of DKA, even in the presence of mildly elevated blood glucose levels. Aggressive intravenous fluid resuscitation should be initiated in patients maintained on canagliflozin, especially in the presence of gastrointestinal illness. Patients who develop canagliflozin-associated DKA should discontinue canagliflozin upon discharge.

## Figures and Tables

**Table 1:** Selected laboratory values from patients 1 and 2.

	Time after presentation (hours)				
	
Patient 1	0	3	6.5	10	12
Sodium (mmol/L)	142	146	148	144	142
Potassium (mmol/L)	4.3	4.2	4.5	4	4.6
Chloride (mmol/L)	102	115	119	117	117
Carbon dioxide (mmol/L)	8*	10*	12*	17	17
Anion gap	32*	21*	17*	10	8
BUN (mg/dL)	43	39	34	31	27
Creatinine (mg/dL)	1.92*	1.31*	1.16*	0.98	0.88
Glucose (mg/dL)	366*	287*	–	–	–
Beta hydroxybutyrate (mmol/L)	12.43*		–	–	–
AST (IU/L)	18	12	–	–	–
ALT (IU/L)	27	18	–	–	–
Lipase (U/L)	164*	–	–	–	–
VBG pH	7.09*	–	–	–	–
VBG pCO_2_ (mmHg)	29*	–	–	–	–
VBG pO_2_ (mmHg)	44	–	–	–	–
VBG HCO_3_ (mmol/L)	8.8*	–	–	–	–
VBG tCO_2_ (mmol/L)	10	–	–	–	–
VBG O_2_ sat (%)	64	–	–	–	–
	Time after presentation (hours)				
Patient 2	0	2.5	5	8	14
Sodium (mmol/L)	137	138	134	134	132
Potassium (mmol/L)	4.7	4.5	4.1	3.9	4.2
Chloride (mmol/L)	102	112	–	111	110
Carbon dioxide (mmol/L)	8*	11*	9*	16	20
Anion gap	27*	15*	–	7	2
BUN (mg/dL)	19	15	12	9	6
Creatinine (mg/dL)	0.76	0.65	0.71	0.62	0.48
Glucose (mg/dL)	327*	249*	334*	235*	103
Beta hydroxybutyrate (mmol/L)	15.35*	–	–	–	–
AST (IU/L)		–	8	–	–
ALT (IU/L)	–	–	8	–	–
Lipase (U/L)	–	–	14	–	–
VBG pH	7.15*	–	7.15*	–	–
VBG pCO_2_ (mmHg)	29*	–	30*	–	–
VBG pO_2_ (mmHg)	81	–	65	–	–
VBG HCO_3_ (mmol/L)	10*	–	10.2*	–	–
VBG tCO_2_ (mmol/L)	11	–	11	–	–
VBG O_2_ sat (%)	92	–	86	–	–

Note:

* Denotes laboratory values of note. BUN, blood urea nitrogen; AST, aspartate transaminase; ALT, alanine transaminase; VBG, venous blood gas.

**Table 2:** Physiologic action of sodium–glucose cotransporters.

	Receptor type	Location	Physiologic action	Antagonism	Illustrative xenibiotics
SGLT-1	Sodium/glucose cotransporter	Small intestine, proximal convoluted tubule	Glucose absorption from gut lumen, reabsorption of glucose from renal filtrate	Glucosuria	Sotagliflozin, phlorizin
SGLT-2	Sodium/glucose cotransporter	Small intestine, alpha cells of pancreas, proximal convolute tubule	Glucose absorption from gut lumen, regulation of glucagon secretion from alpha cells, reabsorption of glucose from renal filtrate	Glucosuria, glucagon secretion from alpha cells	Canagliflozin, sotagliflozin, phlorizin
SGLT-3	G-Protein	Hypothalamus	Signals satiety through regulation of GLP-1, GIP, 5HT	Unknown	–

Note: GLP-1: glucagon-like peptide-1; GIP: glucose-dependent insulinotropic peptide; 5HT: serotonin.
